# The role of pre-treatment and mid-treatment 18F-FDG PET/CT imaging in evaluating prognosis of peripheral T-cell lymphoma-not otherwise specified (PTCL-NOS)

**DOI:** 10.1186/s12880-021-00674-5

**Published:** 2021-10-09

**Authors:** Yafei Zhang, Guangfa Wang, Xin Zhao, Yongxian Hu, Elaine Tan Su Yin, Donghe Chen, Huatao Wang, Kui Zhao

**Affiliations:** 1 Department of PET center, The First Affiliated Hospital, Medical School of Zhejiang University, No. 79 Qingchun Road, Hangzhou, 310003 China; 2 Bone Marrow Transplantation Center, The First Affiliated Hospital, School of Medicine, Zhejiang University, No. 79 Qingchun Road, Hangzhou, 310003 China

**Keywords:** PTCL-NOS, PET/CT, D-5PS, MTV, Prognosis

## Abstract

**Background:**

It has been reported the prognostic value of MTV in predicting the disease prognosis of peripheral T-cell lymphoma (PTCL) through pre-treatment PET/CT imaging. However, these are limited data on pretreatment evaluation and prognosis assessments of peripheral T-cell lymphoma-not otherwise specified (PTCL-NOS). This study aimed to determine the prognostic values of pre-treatment and mid-treatment total metabolic tumor volume (MTV), total lesion glycolysis (TLG), and Deauville 5-Point Scale (D-5PS) in accessing the prognosis of PTCL-NOS.

**Methods:**

A retrospective analysis was conducted in 31 patients with pathologically diagnosed PTCL-NOS. These patients have undergone positron emission PET/CT scanning before and during chemotherapy. Follow-ups were also done to investigate the 2-year progression-free survival (PFS) and Overall Survival (OS) of these patients. During ${}^{18}$F-fluorodeoxyglucose (${}^{18}$F-FDG) PET/CT scans, the MTV and TLG were recorded. Meanwhile, $\bigtriangleup$MTV and $\bigtriangleup$TLG were calculated. Furthermore, the receiver operating characteristic (ROC) curve was employed to classify and to define the threshold values. On the other hand, the mid-chemotherapy assessment and staging of these 31 patients were done by utilizing D-5PS. Subsequently, based on the D-5PS scores obtained, these patients were grouped into two categories: a group of patients with a score of <4 and another group with $\ge$4 points. For these two groups of patients, the survival analysis was done by Kaplan–Meier analysis and a multivariate COX regression model. Moreover, Pearson’s chi-square test ($\chi ^{2}$ test) and Spearman rank correlation coefficient were used to comparing the collected data, respectively.

**Results:**

During the 2-year follow-up period, 15 out of the 31 patients experienced disease progression. The optimal threshold values for both baseline MTV and TLG were 158.16 cm$^{3}$ and 677.40.Additionally, the difference in 2-year PFS between the progressive and non-progressive groups was statistically significant ($\chi ^{2}=8.193$, $P=0.004<0.05$; $\chi ^{2}=8.872$, $P=0.003<0.05$), significant between-group difference was detected for MTV and for TLG ($\chi ^{2}=6.494$, $P=0.011<0.05$; $\chi ^{2}=4.687$, $P=0.03<0.05$). On the other hand, when these patients were classified into two groups according to the mid-chemotherapy Deauville score of <4 and $\ge$4, the statistical difference of 2-year PFS between these two groups was significant, too ($\chi ^{2}=14.966$, $P=0.001<0.05$),but there is no significant between-group difference in OS ($\chi ^{2}=3.337$, $P=0.068<0.05$). COX analysis revealed that D-5PS are the independent factors influencing PFS, while MTV is the independent influencing factor of OS.

**Conclusion:**

The baseline total MTV obtained by PET/CT scanning, and D-5PS are crucial prognostic factors in evaluating the prognosis of PTCL-NOS.

## Background

Peripheral T-cell lymphoma (PTCL) is a relatively rare T-cell non-Hodgkin lymphoma. Several PTCL subtypes include PTCL,not otherwise specified (PTCL-NOS), angioimmunoblastic T-cell lymphoma (AITL), and anaplastic large cell lymphoma (ALCL) [1, 2]. It is worth mentioning that PTCL-NOS is the mostly broad category of PTCL. Most of the PTCLs are aggressive lymphomas with a dismal prognosis. Up till now, the CHOP (cyclophosphamide, doxorubicin hydrochloride (hydroxydaunorubicin), vincristine sulfate (oncovin), and prednisone) regimen is the primary therapy for PTCL [3].

Nonetheless, some of the PTCL patients are resistant to this treatment regimen, which eventually leads to poor clinical outcomes [4, 5]. Moreover, due to resistance to CHOP chemotherapy, the subsequent bone marrow transplantation procedure might also be affected in these patients. Therefore, early identification of chemotherapy-resistant PTCL patients, understanding the chemotherapy response for different PTCL subtypes, establishing better assessment and evaluation methods, and improving individualized treatment regimens are of paramount importance [6, 7].

Generally, PET/CT scan is used for pre-treatment prediction and post-treatment assessment of diffuse large B-cell lymphoma (DLBCL),while over 90% PTCL showed high uptake of F-18 FDG. Several studies have reported the prognostic value of MTV in predicting the disease prognosis of PTCL through pre-treatment PET/CT imaging. Furthermore, it aids in the early detection of high-risk patients with poor prognosis [8]. However, these are only limited data on pretreatment evaluation and prognosis assessments of certain types of PTCL. Thus, the suggested treatment options and therapy guidelines for different PTCL patients are also greatly restricted [3]. The research aimed to focus on the application of parameters such as MTV and TLG in pre-treatment and mid-chemotherapy period to assess the prognosis of PTCL-NOS via PET/CT scans.

## Methods

### Subjects

31 pathologically diagnosed PTCL-NOS patients (18 male patients and 13 female patients) were recruited into this study from January 2014 to January 2016.These patients underwent PET/CT scanning before starting chemotherapy and after 4 courses of the CHOP chemotherapy regimen. Besides, the follow-up of each patient began at the time of confirmed PTCL’s diagnosis via pathology results. The follow-up period ranged from 3 to 70 months, the median follow-up time was 48 months.The characteristics of the patients are listed in Table 1.

**Table 1:** Characteristics of the patients

Factors	Category	Total number of cases	Number of progressive cases	Number of death cases
Age	< 60	23	10	5
	$\ge$ 60	8	5	4
Ann Arbor staging	Stage I–II	10	1	1
	Stage III–IV	21	14	8
IPI Score	Low risk	17	3	1
	(0–2 points)			
	High risk	14	12	8
	(3–4 points)			
SUVmax	< 7.28	14	5	2
	$\ge$ 7.28	17	10	7
MTV (cm$^3$)	< 158.16	15	3	1
	$\ge$ 158.16	16	12	8
D-5PS Score	< 4-Point	21	6	4
	$\ge$ 4-Point	10	9	5

### Imaging techniques

The data acquisition was made by using Siemens PET/CT Biograph 16 Model (Germany), whereas the accelerators system used for F-18 FDG isotopes production was Eclipse Cyclotron with $\ge$98% radiochemical purity. Before the PET/CT examination, the patient was requested to fast for more than 6 h, and the blood glucose level should be controlled below 7.8 mmol/L. Then, F-18FDG was injected intravenously at a prescribed dose of 3.7–4.44 MBq (0.1–0.12 mCi)/kg (according to the patient’s body weight). After 15–20 min of injection, the patient was suggested to take 0.5–1.0 l of water before putting to rest for 60–90 min. Before the test, the patient was asked to empty the bladder. Meanwhile, during the PET/CT scan, the scanning range was from the top of the head to the upper one-third of the thigh. The CT scan parameters were: Voltage 120 kV; current 150 mA; Slice thickness: 5 mm, while the PET emission scan was acquired for 5 min/bed (skull) and 2 min/bed (trunk).

### Medical image analysis

The PET imaging data were reviewed by two experienced nuclear medicine physicians to interpret the lesions in cross-sectional, coronal, and sagittal planes. Siemens ESOFT workstation was used to analyze images, and it was thresholding at SUV $=$ 2.5. Then, the region of interest (ROI) was outlined, while the maximum standardized uptake value (SUVmax) and the mean standardized uptake value (SUVmean) were automatically obtained via the ROI calculations. Simultaneously, the metabolic tumor volume (MTV) before and during chemotherapy were also defined. In the data interpretation, the pre-treatment PET/CT baseline SUVmax was expressed as SUVmax$_0$, whereas the SUVmax obtained amid chemotherapy treatment (after 4 courses of CHOP chemotherapy) was expressed as SUVmax$_1$. Thus, the difference in the SUVmax values was expressed as:

1 $$\begin{aligned} \varDelta SUV\,max= \left( {\frac{SUV\,max_{0}-SUVmax_{1}}{SUV\,max_{0}}} \right) \end{aligned}$$

Furthermore, the pre-treatment baseline MTV was expressed as MTV$_0$, while the MTV obtained during chemotherapy was represented by MTV$_1$. Hence, the difference in the $\bigtriangleup$MTVs was expressed as:

2 $$\begin{aligned} \varDelta MTV= \left( {\frac{MTV_{0}-MTV_{1}}{MTV_{0}}} \right) \end{aligned}$$

Next, the baseline TLG of pre-treatment PET/CT was marked as TLG$_0$. Meanwhile, TLG$_1$ was the TLG obtained in mid-chemotherapy PET/CT scans (after 4 courses of chemotherapy). The $\bigtriangleup$TLG was calculated by:

3 $$\begin{aligned} \varDelta TLG= \left( {\frac{TLG_{0}-TLG_{1}}{TLG_{0}}} \right) \end{aligned}$$

On the other hand, the total lesion glycolysis (TLG) was calculated by the formula below:

4 $$\begin{aligned} TLG= SUV\,mean\times MTV. \end{aligned}$$

### Evaluation criteria

The Deauville 5-Point Scale (D-5PS) was used as one of the evaluation standards in this study. The scoring system is as stated below (Table 2).

**Table 2:** The Deauville 5-point scoring system

Score	F-18 FDG uptake
1	No uptake or no residual lesion
2	The uptake of residual lesions $\le$ mediastinal blood pool uptake
3	Mediastinal blood pool uptake $\le$ residual lesion uptake < liver uptake
4	The residual lesion uptake is slightly higher than liver uptake
5	The residual lesion uptake is 2–3 times more than liver uptake or new lesions

During the follow-up period, the treatment responses of these patients were evaluated according to newly revised criteria for lymphoma’s treatment evaluation by the International Working Group. Based on the treatment responses, they were classified into the following categories, including complete remission (CR), partial remission (PR), stable disease (SD), and relapsed disease or progressive disease (PD). Apart from the criteria mentioned above, the 2-year progression-free survival (PFS) was another indicator of treatment efficacy. By 2-year PFS, the patients were further divided into progressive disease and non-progressive disease groups. The follow-up would be discontinued if the patients had disease progression, recurrence, and death within these two years, or after the two-year follow-up visits.

The overall survival refers to the time interval from the start of treatment to the last follow-up or death from any cause. Based on the results from follow-up, the patients were divided into survival group and death groups.

### Data processing/analysis

The statistical analysis was carried out using SPSS 18.0 Software. The data which conformed to a normal distribution were analyzed by ANOVA or t-test, while non-normally distributed data were compared by non-parametric tests. Next, ROC curve analysis was performed for patient groupings and to determine the optimal cutoff value for SUVmax, MTV, and TLG. The PFS and OS have been taken as dependent variables and SUVmax, MTV and TLG before chemotherapy have been taken as independent variables, the cutoff values were found after drawing the ROC curve.In the same way, the cutoff values of $\bigtriangleup$SUVmax, $\bigtriangleup$MTV and $\bigtriangleup$TLG were found, respectively.

Furthermore, Kaplan–Meier curves and Cox regression analysis were utilized to investigate the relationships between the parameters and 2-year PFS or OS.

### Data output/interpretation

The kappa statistic was done in both observers for test score consistency. Besides, patients were grouped into two groups, with Deauville score of 4 as the threshold value. Then, the data comparison between groups was analyzed by Kaplan–Meier survival analysis and Cox regression analysis. Moreover, these patients were also being classified into high-risk and low-risk groups, in accord with the scoring system of the National Comprehensive Cancer Net-International Prognostic Index (NCCN-IPI scores). Subsequently, the Kaplan–Meier curve and Cox regression analysis were employed to analyze the data, whereas the Spearman correlation test was performed on the Deauville score and MTV. A *p* value of less than 0.05 ($P<0.05$) indicated strong evidence of statistical significance.

## Results

### Pre-treatment assessment and general information of patients

In this study, 31 patients were grouped and scored according to NCCN-IPI before treatment. The results showed that 14 of these patients were categorized as high-risk patients and 17 low-risk patients. Among these patients, 10 patients were in Ann Arbor Stage I–II, and another 21 of them were in Ann Arbor Stage III–IV. Within the two years of follow-ups, 15 cases (48.39$\%$) had disease progression. The rate of PFS and OS was 51.61$\%$ and 70.97$\%$, respectively. The outcomes of the non-parametric tests were displayed in Table 1.

### Pre-treatment and post-treatment data analysis of SUVmax, MTV and TLG

While there was no statistical difference in SUVmax ($z=-0.909$, $P=0.379 >0.05$) between progressive and non-progressive groups, the MTV ($z=-2.925$, $P=0.003<0.05$) and TLG ($z=-0.269$, $P=0.007<0.05$) of these two groups exhibited statistical significance. What is more, the $\bigtriangleup$SUVmax ($z=-1.381$, $P=0.167 >0.05$) ,$\bigtriangleup$MTV ($z=-1.304$, $P=0.192>0.05$), and $\bigtriangleup$TLG ($z=-1.344$, $P=0.179 >0.05$) analyses before therapy and after four courses of chemotherapy showed no statistical difference between the progressive and non-progressive groups, too. There are no significant differences in SUVmax and TLG between the survival and the death groups ($z=-0.87$, $P=0.403>0.05$; $z=-1.784$, $P=0.078>0.05$), while significant difference in MTV was detected ($z=-0.2002$, $P=0.046<0.05$). Also, results calculated based on the data before and after four cycles of chemotherapy on the comparison of patients within the survival and the death groups showed no significant differences in $\bigtriangleup$SUVmax ($z=-0.392$, $P=0.716>0.05$), $\bigtriangleup$MTV ($z=-1.567$, $P=0.124>0.05$) and $\bigtriangleup$TLG ($z=-1.48$, $P=0.147>0.05$).

### ROC curve and Kaplan–Meier survival analysis

**Fig. 1:**
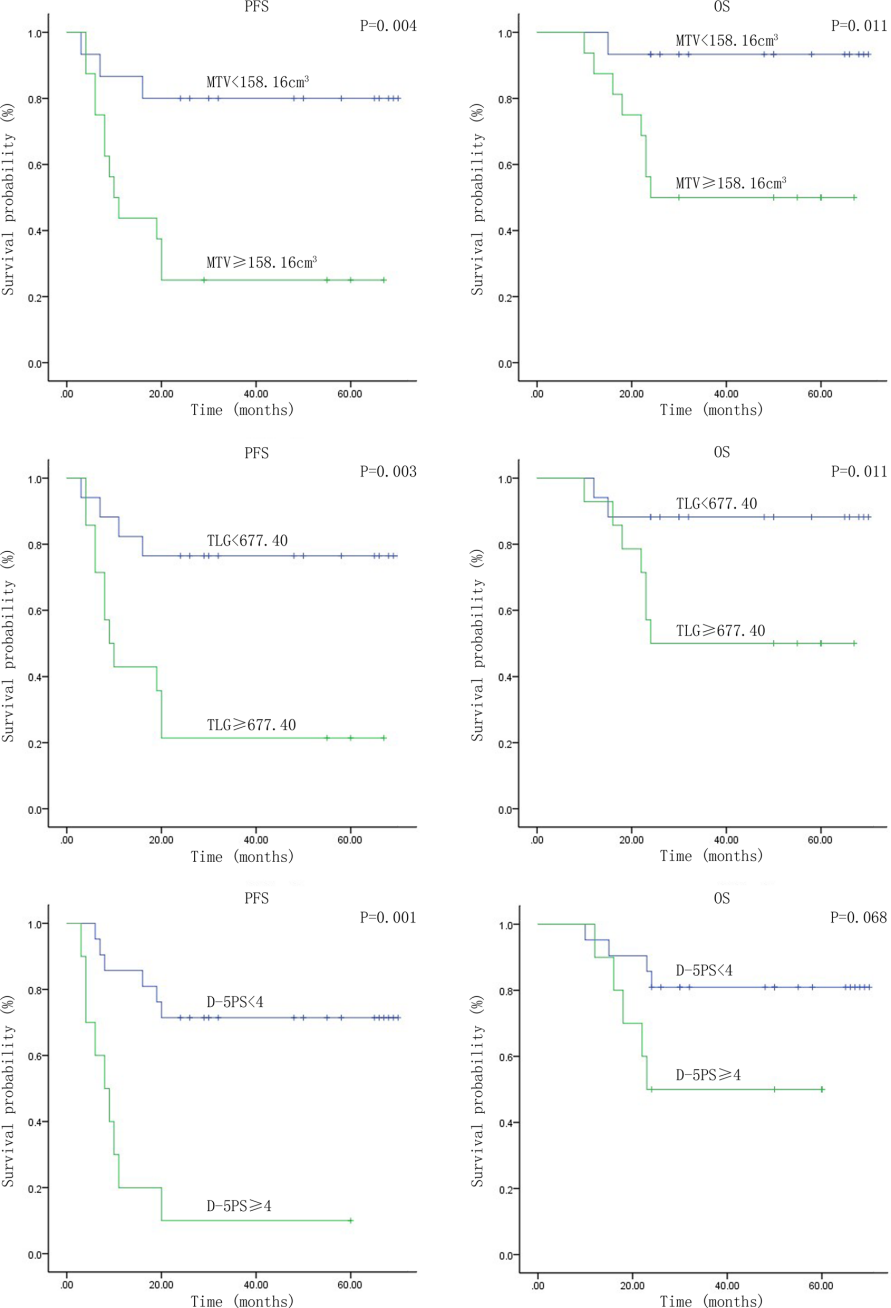

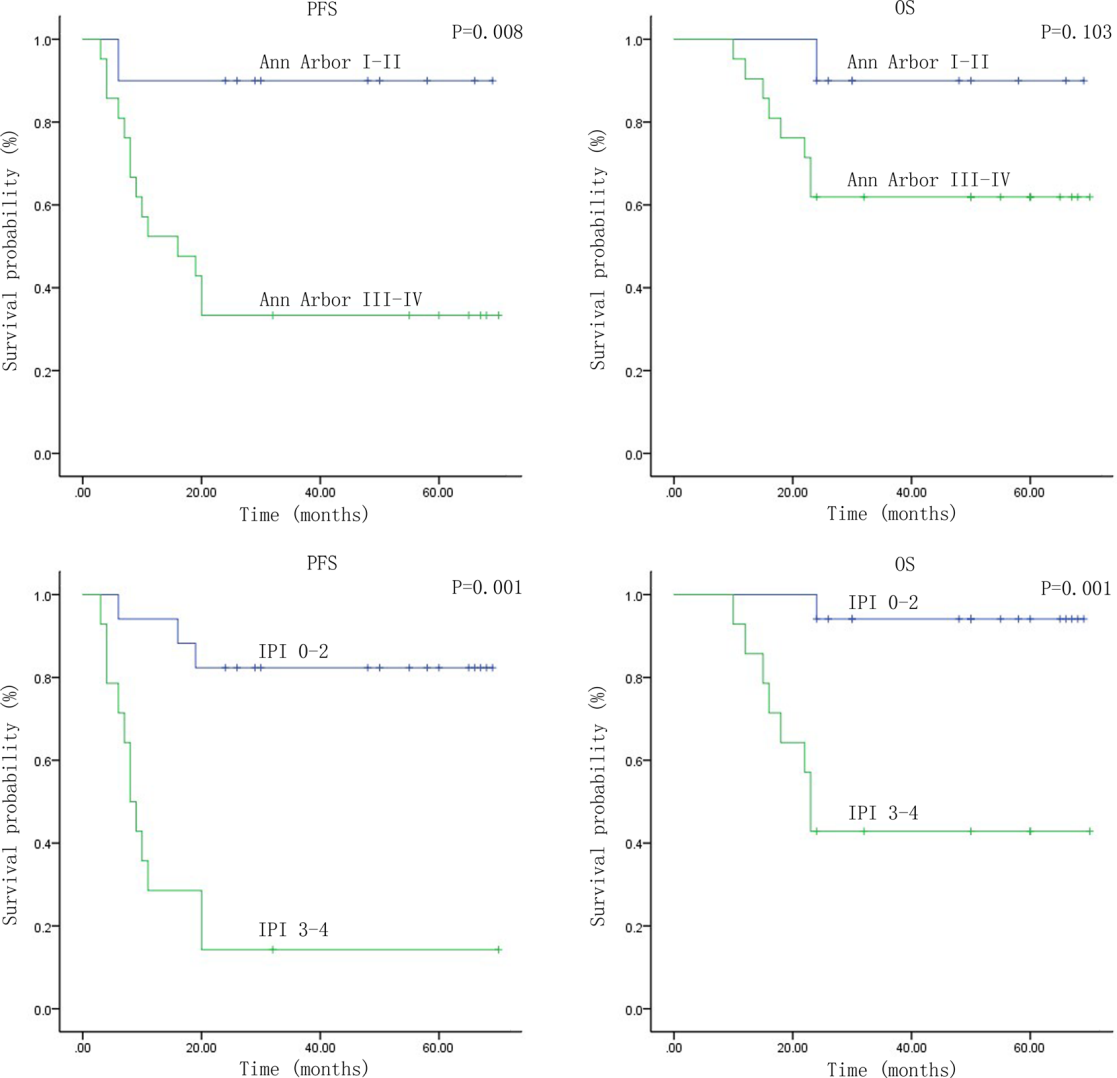
Kaplan–Meier survival curves for progression-free survival (PFS) and overall survival (OS) in patients with PTCL-NOS, including MTV, TLG, D-5PS, Ann Arbor and IPI

The ROC curve was used to determine and calculate the optimal thresholds or cutoff values of pre-treatment SUVmax, MTV, and TLG in order to estimate PFS and OS. The patients were then divided into two groups according to the thresholds given. The 2-year PFS survival analyses between these two groups were executed by applying Kaplan-Meier curves. In this section, the groupings were first done with the cutoff values of SUVmax $=$ 7.28, MTV $=$ 158.16 cm$^3$, and TLG $=$ 677.40. As a result, the 2-year PFS difference was not statistically significant when the groups were compared at SUVmax $=$ 7.28 ($\chi ^{2}y=1.004$, $P=0.316>0.05$). Nevertheless, the 2-year PFS difference was statistically significant between groups when MTV $=$ 158.16 cm$^3$($\chi ^{2}=8.193$, $P=0.004 <0.05$) and TLG $=$ 677.40 ($\chi ^{2}=8.772$, $P=0.003<0.05$). Besides, the groups formed when $\bigtriangleup$SUVmax = 81.33$\%$, $\bigtriangleup$MTV $=$ 54.7$\%$, and $\bigtriangleup$TLG=71.9$\%$ were also being compared. There were no statistical significances in 2-year PFS for the groups with thresholds of $\bigtriangleup$SUVmax=81.33$\%$ ($\chi ^{2}=0.494$, $P=0.482>0.05$), $\bigtriangleup$MTV $=$ 54.7$\%$ ($\chi ^{2}=0.083$, $P=0.774>0.05$) and $\bigtriangleup$TLG $=$ 71.9$\%$ ($\chi ^{2}= 0.115$, $P=0.734>0.05$). Then using SUVmax $=$ 7.28 as the cutoff for dividing the patients into two groups, there were no statistical significances in OS($\chi ^{2}=2.092$, $P=0.148 >0.05$). Using MTV $=$ 158.16 cm$^3$, TLG $=$ 677.40 as the cutoff for dividing the patients into two groups, there were statistical significances in OS for the groups($\chi ^{2}=6.494$, $P=0.011<0.05$, $\chi ^{2}=4.687$, $P=0.03<0.05$). In addition, There were no statistical significances in OS for the groups with thresholds of $\bigtriangleup$SUVmax=81.33$\%$ ($\chi ^{2}=0.301$, $P=0.583>0.05$), $\bigtriangleup$MTV $=$ 54.7$\%$ ($\chi ^{2}=1.143$, $P=0.285>0.05$) and $\bigtriangleup$ TLG $=71.9\%$ ($\chi ^{2}=0.31$, $P=0.86>0.05$) (Fig. 1).

### The deauville 5-point scale (D-5PS) and survival analysis

The D-5PS scorings of the patient’s treatment response after 4 cycles of CHOP chemotherapy regimens were done by two experienced nuclear medicine physicians. The D-5PS scorers made consistent judgments across all tests(Kappa $=0.503$, $P<0.05$).The Kaplan–Meier curves (Fig. 1) illustrated the PFS of the two groups of patients with D-5PS < 4 and $\ge$4, suggesting a statistical difference between the groups ($\chi ^{2}=14.996$, $P=0.001<0.05$), and the OS difference was not statistically significant ($\chi ^{2}=3.337$, $P=0.068>0.05$). The Kaplan–Meier survival analysis depicted a statistically significant difference in 2-year PFS between the low-risk group (IPI 0-2 point) and the high-risk group (IPI 3-4 point) ($\chi ^{2}=16.346$, $P=0.001<0.05$),while no significant difference in OS between the two groups ($\chi ^{2}=10.767$, $P=0.001<0.05$).Next, the 2-year PFS and OS difference between Ann Arbor Stage I–II and Stage III–IV groups were also statistically significant ($\chi ^{2}=7.023$, $P=0.008<0.05$; $\chi ^{2}=2.66$, $P=0.103<0.05$). Furthermore, when the high-risk patients in NCCN-IPI and the Ann Arbor Stage III–IV patients were further categorized by Deauville scores of < 4 and $\ge$ 4, the differences in 2-year PFS were statistically significant ($\chi ^{2}=6.734$, $P=0.009<0.05$, $\chi ^{2}=6.76$, $P=0.009<0.05$).

### COX regression analysis and correlation analysis of pre-chemotherapy MTV and deauville 5-point scale (D-5PS)

Univariate and multivariate COX regression analyses were respectively carried out on the factors like Ann Arbor staging, IPI score, SUVmax, baseline MTV, and Deauville score. The single-factor analysis of variance showed that Ann Arbor staging, NCCN-IPI, baseline MTV, and Deauville score were the significant predictors of 2-year PFS. Nonetheless, the multiple factor analysis indicated that only the D-5PS score was the only key factor (Table 3). Univariate Cox hazard analysis showed that baseline MTV and Ann Arbor staging significantly predicted 2-year OS, multivariate analysis showed that only baseline MTV was a significant factor (Table 4). Furthermore, a positive correlation was shown between baseline MTV and Deauville score in Spearman correlation analysis ($r=0.53$, $P=0.002<0.05$). Meanwhile, the Deauville score was positively correlated with both Ann Arbor staging and NCCN-IPI score ($r=0.575$, $P=0.001<0.05$, $r=0.378$, $P=0.036<0.05$).The baseline MTV was positively correlated with Ann Arbor staging and NCCN-IPI ($r=0.437$, $P=0.014<0.05$; $r=0.360$, $P=0.047<0.05$).

**Table 3:** COX regression analyzes The factors predictive of survival associated with PFS in patients with PTCL-NOS on univariate and multivariate analysis

Factors	Single-factor analysis		Multiple-factor analysis	
	*P* value	HR (95% CI)	*P* value	HR (95% CI)
Ann Arbor staging	0.031	9.333 (1.222–0.901)	0.359	3.189 (0.267–38.015)
IPI Score	0.001	0.118 (0.033–0.423)	0.754	1.354 (0.203–9.026)
SUVmax	0.751	1.013 (0.936–1.096)	0.059	0.866 (0.745–1.006)
Pretreatment MTV (cm3)	0.015	0.205 (0.057–0.731)	0.117	1.001 (1.000–1.002)
D-5PS Score	0.001	0.166 (0.058–0.481)	0.01	2.482 (1.245–4.946)

**Table 4:** COX regression analyzes the factors predictive of survival associated with OS in patients with PTCL-NOS on univariate and multivariate analysis

Factors	Single-factor analysis		Multiple-factor analysis	
	*P* value	HR (95% CI)	*P* value	HR (95% CI)
Ann Arbor staging	0.141	0.21 (0.026–1.682)	0.947	0.0 (0.0–3.82)
IPI Score	0.013	14.284(1.769–14.742)	0.94	3.26 (0.00–8.01)
SUVmax	0.734	1.018 (0.917–1.130)	0.312	0.933 (0.817–1.067)
Pretreatment MTV (cm3)	0.010	1.002 (1.000–1.003)	0.038	28.102 (1.206–654.603)
D-5PS Score	0.041	1.648 (1.02–0.2.663)	0.184	0.565 (0.243–1.312)

### The data analysis of deauville scores and baseline MTV

The patients were grouped based on the combination of both factors, Deauville scores, and baseline MTV. It was aimed to calculate the percentage of 2-year progression-free survival (PFS) among these patients. The results were as follows: (a) For the group with a Deauville score <4 and MT <158.16 cm$^3$, the 2-year PFS accounted for 85.71$\%$ (12/31). (b) For the group with a Deauville score <4 and MTV $\ge$158.16 cm$^3$, the 2-year PFS accounted for 42.86$\%$ (3/31). (c) For the group with a Deauville scor $\ge$4 and MTV <158.16 cm$^3$, the 2-year PFS accounted for 0.00$\%$ (0/31). (d) For the group with a Deauville score$\ge$4 and MTV $\ge$158.16 cm$^3$, the 2-year PFS accounted for 11.11$\%$ (1/31).The group with the worst prognosis of PFS accounted for 3.22$\%$ of the total number of patients but allocated 11.11$\%$ among the total number of progressive patients.

## Discussion

The role of ${}^{18}$F-FDG PET/CT before and during chemotherapy is indispensable for the post-treatment evaluation of DLBCL. Other than DLBCL, several studies have also indicated that PET/CT scanis useful in predicting early recurrence and poor prognosis in PTCL. It is proved that mid-chemotherapy PET/CT scan is beneficial in evaluating treatment response and predicting the prognosis of PTCL. For instance, the patients with negative findings in PET/CT scans tend to have much improved 2-year PFS and OS as compared to those with positive findings, but the results remain controversial [9, 10]. Up till today, factors like SUVmax, MTV, and Deauville score are the most used prognostic indicators for PTCL’s prognosis prediction. However, PTCL-NOS, as the most common pathological subtype of PTCL, is still lacking standard criteria for early evaluation and prognosis prediction.

Clinically, high ${}^{18}$F-FDG uptake on PET/CT is often considered as an essential marker for aggressive NHL. Previous studies have found that SUV>10 in PET/CT imaging may predict lymphomas with aggressive histology [11]. In this study, 31 patients with PTCL-NOS were followed up for 2 years. The results revealed that the SUVmax difference obtained from the progressive and non-progressive groups or the survival and death groups was not statistically significant ($P>0.05$).This finding further validated that $\bigtriangleup$SUVmax might not be a good indicator for early prediction of the disease. The possible explanations for SUVmax unreliability are mainly related to the high invasiveness and poor prognosis of PTCL-NOS. Moreover, SUVmax is easily influenced by the injection of contrast agents, time or parameters of PET/CT acquisitions, and the patient’s blood glucose level. Also, SUVmax with a lower value might affect its prognostic value [12].

The pre-chemotherapy baseline MTV and TLG have been reported as the prognostic factors of PTCL. According to the earlier studies, higher MTV and TLG values are indicative of poor prognosis and predisposition to earlier relapse/recurrence or shorter survival [13]. Furthermore, the baseline MTV and TLG usually reflect the actual general conditions of the patients prior to chemotherapy. Our study has also manifested that the category with lower baseline MTV and TLG would have a better 2-year PFS relative to the high baseline MTV and TLG group ($\chi ^{2}=9.378$, $P=0.002<0.05$, $\chi ^{2}=9.314$, $P=0.002<0.05$), besides, low baseline MTV or TLG values relate with better overall survival, which also indicated PTCL-NOS prognosis. This outcome has also explained that the baseline MTV and TLG might be the best substitutes for those patients who are not suitable to undergo a prognostic assessment by using IPI score and Ann Arbor staging. It also indicated that $\bigtriangleup$MTV and $\bigtriangleup$TLG at baseline PET/CT imaging and during chemotherapy are significant in the prognostic evaluation of PTCL-NOS as the tumor size prior to treatment and the levels of tumor glycolysis are closely related to its prognosis.

Similarly, the previous studies have also shown that $\bigtriangleup$MTV at baseline and mid-chemotherapy are feasible to the prognostic evaluation of PTCL [14]. However, in the 2-year follow up period of these PTCL-NOS patients, the data which obtained from progressive and non-progressive patients showed no significant difference. Additionally, by setting the cutoff values of $\bigtriangleup$MTV $=$ 54.7$\%$ and $\bigtriangleup$TLG=71.9$\%$, the survival analyses also suggested that the 2-year PFS and OS between these groups were not statistically significant ($P>0.05$). These findings proposed that neither of these parameters has remarkable effects in determining progression-free survival and predicting prognosis of PTCL-NOS patients, which might imply that the predictability of chemotherapy response for prognosis is limited. Perhaps, the prognostic index of PTCL-NOS is somewhat different from the regular PTCL criteria, requiring independent prognostic indicators for PTCL-NOS instead. Then, $\bigtriangleup$MTV and $\bigtriangleup$TLG represented the effect and post-treatment changes through quantifying tumor glycolysis and the tumor size before and after treatment. Nonetheless, their infeasibility in estimating disease prognosis may be related to the treatment duration and the tumor sensitivity to chemotherapeutic drugs.

Our results demonstrated that, baseline MTV, but not TLG, was significantly differed between the survival and the death groups. In line with previous reports [15], Kaplan–Meier in the current work showed that patients with both low MTV and TLG levels are with better OS. This indicated that the relationship between TLG and OS is not simply causal, but might further relate with the effect of SUVmax on the amount of glycolysis. Notwithstanding, the results of OS may also affected by other factors. Also, in compliance with previous studies on the relationship with PTCL, it was here found that the MTV which differed between the survival and the death groups was the independent influencing factor of OS. This indicated that MTV could effectively predict the survival of PTCL-NOS, and hence as a meaningful index for prognosis.

Besides MTV and TLG, the Deauville 5-Point Scale (D-5PS)is also one of the most widely used indicators for prognostic prediction of DLBCL. To prove, D-5PS was used for the assessment of PTCL-NOS in our study, too.In this study, a positive result was achieved when the Deauville score was $\ge$4 (more than the liver uptake value). Overall, patients with positive results had a lower 2-year PFS than negative (D-5PS<4) patients ($\chi ^{2}=10.236$, $P=0.001<0.05$). The positive results allocated approximately 32.26$\%$ of the patients, suggesting that some PTCL-NOS patients have not achieved complete remission (CR), while some of them were not sensitive or responding to CHOP regimens. These treatment responses might significantly influence treatment decision-making. Therefore, these patients were further stratified by the combination of baseline MTV and D-5PS. As a result, the group with the worst prognosis (a Deauville score$\ge$4 and MTV$\ge$158.16 cm$^3$) with progression-free took up 3.22$\%$ of the total patients within 2 years, yet 11.11$\%$ of the total progressive cases. This approach seemed to make it easier to screen and identify high-risk patients, and thus, it may predict prognosis and the likelihood of early relapse more accurately.

In addition to the parameters mentioned earlier, NCCN-IPI is used clinically to assess patients with NHL and predict treatment outcomes, though its clinical significance in patients with PTCL remains controversial [16, 17].In this research, there was a significant statistical difference in 2-year PFS between patients in the low-risk group and those in the high-risk group ($P=0.001<0.05$). Consequently, the NCCN-IPI score might be relevant to the prognosis of PTCL-NOS. Next, the high-risk patients were been grouped again by the Deauville Five-Point Scale. There,a statistical difference was observed in 2-year PFS between these groups ($\chi ^{2}=6.734$, $P=0.009<0.05$). At the same time, this research discovered that Ann Arbor staging exerted an effect on the 2-year PFS of these 31 PTCL-NOS patients, unlike the previous studies on PTCL. The prognostic assessments by Ann Arbor staging are usually not affected by tumor size and metabolism. Also, Ann Arbor staging is widely applied to almost all pathological subtypes. Later, the patients were categorized with III–IV stage in Ann Arbor classification with D-5PS.There was a statistical difference in 2-year PFS between groups ($\chi ^{2}=6.76$, $P=0.009<0.05$). It proved that the sub-stratification by the Deauville 5-point Scale is beneficial for clinically high-risk patients. Furthermore, together with the clinical-guiding significance, it alters the risk stratification of clinical patients. In short, based on the analyses, the IPI score and Ann Arbor staging are beneficial for larger groups in clinical prediction, whereas the Deauville score is more representative of individual response to treatment.

Generally, the evaluation and prediction of PTCL prognosis by PET/CT scan mainly involved SUV, MTV, TLG, Deauville score. Given the limited sample size in this study, the COX regression analysis could only be performed based on three parameters, namely SUVmax, pre-chemotherapy baseline MTV, Deauville-5PS, and another two staging systems like IPI risk stratification and Ann Arbor staging. In this present study, the single-factor analysis (ANOVA)exhibited that Ann Arbor staging, NCCN-IPI, prechemotherapy MTV, and Deauville score were the feasible prognostic factors of 2-year PFS in PTCL-NOS patients, while D-5PS had independent predictive value for 2-year PFS. It is worth noticing that the PET/CT Deauville score is confined to the visual-based assessment and subjective evaluation by different scorers. Thus, it is one of the limitations of this study. However, the Kappa test was used to overcome this limitation in order to ensure the consistency of the results given by two nuclear physicians. Through the analysis, It has been assured that the results are somewhat reliable and consistent. Hence, D-5PS can be used as an indicator of disease progression for PTCL-NOS patients.

Moreover, the further correlation analysis in this study depicted a positive correlation between pre-chemotherapy MTV and Deauville score ($r=0.483$, $P=0.006<0.05$). This outcome suggested that the tumor size before chemotherapy would have an impact on mid-treatment imaging, which might also be essential for prognostic assessment and prediction. Furthermore, the Deauville score was found to be positively correlated with Ann Arbor staging and NCCN-IPI scores, too. The positive correlations between these indicators further validated that PET/CT is useful in the assessment of PTCL-NOS. It is also associated with clinical manifestations and risk levels of the disease. Thus, this study summarized that the Ann Arbor staging and NCCN-IPI scores are both significant for NHL treatment. Nevertheless, it is mostly derived from the large sample size and lacking specificity and individualized criteria. Based on this study, it could be deduced that the ${}^{18}$F-FDG PET/CT scan with the aforementioned parameters would be a better prognosis evaluation method for PTCL-NOS. Therefore, the treatment plans for PTCL-NOS should be improved based on prognostic assessment of disease recurrence and progression.The measurement of pre-treatment MTV can guide the pre-treatment process of tumor therapy and also guide the course of treatment, such as stem cell transplantation and immunotherapy at the early stage of treatment.In the middle stage of treatment, this is a common phenomenon that the tumor metabolism has been reduced when tumor shrinkage is not obvious.In the middle stage of treatment, this is a common phenomenon that the tumor metabolism has been reduced when tumor shrinkage is not obvious.When there is necrosis and fibrosis in the tumor site of some patients, the morphology will not disappear completely, at this time, the tumor metabolism has disappeared which means clinical recovery.In clinical practice, the decision to terminate treatment can be made timely after the evaluation of indicators such as D-5PS, so as to improve patients’ own benefits, improve economic benefits and reduce unnecessary treatment.

Certainly, there are still some limitations in this study. For example, the short follow-up period for overall survival (OS), this shortcoming should be improved in further studies,and the follow-up will be continued for the survival period.In addition, the number of patients still limits the impact of various parameters on disease survival, making the results imperfect. Moreover, the research did not more exploration on the significance of prognosis prediction in treatment.

## Conclusion

In conclusion, the PET/CT scan’s baseline metabolic tumor volume (MTV), total lesion glycolysis (TLG), and Deauville 5-Point Scale (D-5PS) are highly valuable and feasible to determine mid-chemotherapy treatment response in patients with PTCL-NOS. These factors are significant for prognosis prediction in PTCL-NOS patients, too. Additionally, the combination model of the prognostic factors like MTV and D-5PS outperforms the single-factor model in assessing the prognosis of PTCL-NOS patients. Meanwhile, for patients at clinically high-risk, Deauville 5-Point Scale is more useful in predicting the prognosis of different patients, baseline MTV could better predict patient survival.

## Data Availability

The datasets used and/or analysed during the current study are available from the corresponding author on reasonable request.

## Abbreviations

PTCL: Peripheral T-cell lymphoma
PTCL-NOS: Peripheral T-cell lymphoma-not otherwise specified
MTV: Metabolic tumor volume
TLG: Total lesion glycolysis
D-5PS: Deauville 5-Point Scale
PFS: Progression-free survival
OS: Overall survival
